# UBC^®^ *Rapid* Test—A Urinary Point-of-Care (POC) Assay for Diagnosis of Bladder Cancer with a focus on Non-Muscle Invasive High-Grade Tumors: Results of a Multicenter-Study

**DOI:** 10.3390/ijms19123841

**Published:** 2018-12-02

**Authors:** Thorsten H. Ecke, Sarah Weiß, Carsten Stephan, Steffen Hallmann, Christian Arndt, Dimitri Barski, Thomas Otto, Holger Gerullis

**Affiliations:** 1 HELIOS Hospital, Department of Urology, Bad Saarow D-15526, Germany; steffen.hallmann@helios-gesundheit.de; 2 Department of Urology, Charité University Hospital, Berlin D-10117, Germany; sarah.weiss2@helios-gesundheit.de (S.W.); carsten.stephan@charite.de (C.S.); 3 Berlin Institute for Urological Research, Berlin D-10115, Germany; 4 Department of Urology, Lukas Hospital Neuss, Neuss D-41464, Germany; arndt_christian@web.de (C.A.); barskidimitri@gmail.com (D.B.); thomas_otto@lukasneuss.de (T.O.); 5 University Hospital for Urology, Klinikum Oldenburg, School of Medicine and Health Sciences Carl von Ossietzky University Oldenburg, Oldenburg D-26133, Germany; holger.gerullis@gmx.net

**Keywords:** bladder cancer, tumor markers, urinary based diagnostics

## Abstract

Objectives: UBC^®^ *Rapid* Test measures soluble fragments of cytokeratins 8 and 18 in urine. We present results of a multicenter study using an updated version of UBC^®^ *Rapid* Test in bladder cancer patients, patients with urinary bladder cancer positive history, and healthy controls. Material and Methods: In total 530 urine samples have been included in this study. Clinical urine samples were used from 242 patients with tumors of the urinary bladder (134 non-muscle-invasive low-grade tumors (NMI-LG), 48 non-muscle-invasive high-grade tumors (NMI-HG), and 60 muscle-invasive high-grade tumors (MI-HG)), 62 patients with non-evidence of disease (NED), and 226 healthy controls. Urine samples were analyzed by the UBC^®^ Rapid point-of-care (POC) assay and evaluated by Concile Omega 100 POC Reader. All statistical analyses have been performed using R version 3.2.3. Results: Elevated levels of UBC^®^ Rapid Test in urine are higher in patients with bladder cancer in comparison to the control group (*p* < 0.001). The sensitivity for the whole bladder cancer cohort was 53.3% (positive predictive value (PPV) 90.2%, negative predictive value (NPV) 65.2%) and was 38.8% (PPV 78.8%, NPV 72.1%) for non-muscle-invasive low-grade bladder cancer; 75.0% (PPV 72.0%, NPV 94.7%) for non-muscle-invasive high-grade bladder cancer and 68.3% (PPV 74.6%, NPV 91.8%) for muscle-invasive high-grade bladder cancer. The specificity for the statistical calculations was 93.8%. The cut-off value (10 µg/L) was evaluated for the whole patient cohort. The area under the curve of the quantitative UBC^®^ Rapid Test using the optimal threshold obtained by receiver operating characteristics (ROC) analysis was 0.774. Elevated values of UBC^®^ *Rapid* Test in urine are higher in patients with high-grade bladder cancer in comparison to low-grade tumors and the healthy control group. Conclusions: UBC^®^ *Rapid* Test has potential to be a clinically valuable urinary protein biomarker for detection of high-grade bladder cancer patients and could be added in the management of NMI-HG tumors. UBC^®^ *Rapid* results generated in both study centers in the present multicenter study are very similar and reproducible. Furthermore UBC^®^ *Rapid* Test is standardized and calibrated and thus independent of used batch of test as well as study site.

## 1. Introduction

In Europe bladder cancer (BCa) is the fifth most frequent cancer. Its incidence rate was 151,200 and its annual mortality rate was 51,400 cases in 2012 [1]. Around 30% of bladder cancer patients suffered from muscle-invasive bladder cancer (MIBC) at the time of first diagnosis [2]. Radical cystectomy (RC) is the gold standard to treat patients with MIBC.

Non-muscle invasive high-grade bladder cancer has a particularly high rate of recurrence and will progress to muscle-invasive disease. The ideal urine-soluble marker should be used for primary diagnosis, follow-up, and screening of high-risk populations; replacing cystoscopy during follow-up or decreasing the number of control cystoscopies during follow-up would be a worthwhile goal. Due to its contact with urine, malignant cells are shed into the urine, and this urine contains the carcinogens producing the malignancy. Some of these urinary based tests have a higher specificity and sensitivity than classical urine cytology and could be important for screening and case findings [3].

Intermediate filaments of the cytoskeleton of epithelial cells containing cytokeratins are often overexpressed in urothelial tumors. In humans twenty different cytokeratins have been identified, and cytokeratins 8, 18, and 19 are known to be important in urothelial cells [4]. The expressions of cytokeratins such as 8, 18, and 19 are higher in urothelial cells and may be elevated because of a higher cell turnover rate [5,6]. Immunohistochemical features of urothelial dysplasia include aberrant cytokeratin 20 expression at different levels of the urothelium, however, there is also usually overexpression of p53 and high Ki-67 index [7].

UBC^®^ *Rapid* Test is based upon an immunochromatographic method and measures fragments of cytokeratin 8 and 18 qualitatively. The measured levels are lower in low-grade tumors and benign urological diseases [8,9]. Cytokeratins 8 and 18 are soluble in urine and can be detected quantitatively with monoclonal antibodies using sandwich ELISA as well as UBC^®^ *Rapid* assay with a photometric reader. It is important to highlight that this version of UBC^®^ *Rapid* Test is a modified and updated version of fast cytokeratin determination in urine in comparison to the assay introduced 15 years ago. Furthermore this new version of UBC^®^ *Rapid* Test is used in combination with a reader to quantitate the signal quite comparably with an ELISA assay, but it is a point-of-care (POC) assay. Previous UBC Rapid assays were only assays for visual evaluation of results.

In the last publication of our group we had a focus on carcinoma in situ (CIS), and we could show excellent results for UBC^®^ *Rapid* Test for detecting CIS [10]. Regarding these facts, it is mandatory to include new tests into bladder cancer diagnostics, specifically a test that could detect flat, high-risk tumors difficult to detect in cystoscopy would be a step to ameliorate the finding of these tumors. The aim of this multicenter study is to report the final results with the highest number of measured samples for UBC^®^ *Rapid* Test and to evaluate the usefulness of UBC^®^ *Rapid* Test in patients with urinary bladder cancer with a focus on non-muscle invasive high-grade (NMI-HG) tumors and compare with healthy individuals.

## 2. Results

A total of 530 patients were included in the study; 242 with confirmed bladder cancer, 62 with non-evidence of disease (NED), and 226 healthy controls with no history of bladder cancer. The median age of the study population was 73 (range 26–98) years. Of these patients, 391 (73.8%) were men and 139 (26.3%) were women. Among the 242 patients with confirmed bladder cancer, 134 had non-muscle-invasive low-grade (NMI-LG), 48 had NMI-HG, and 60 had muscle-invasive high-grade (MI-HG) BCa; 182 (75.2%) had non-muscle-invasive bladder cancer (pTa and pT1 tumors), 60 (24.8%) had stage pT2–4. Carcinoma in situ (CIS) was detected in 23 cases (9.5%). A detailed analysis of the CIS patients in this study had already been published [10].

The number of patients and healthy controls are listed in Table 1 for study center I (HELIOS Hospital Bad Saarow) and study center II (Lukaskrankenhaus Neuss). Both groups enrolled a similar number of patients in the study. Table 2 shows all relevant data for center 1 and center 2 separately.

**Figure 1:**
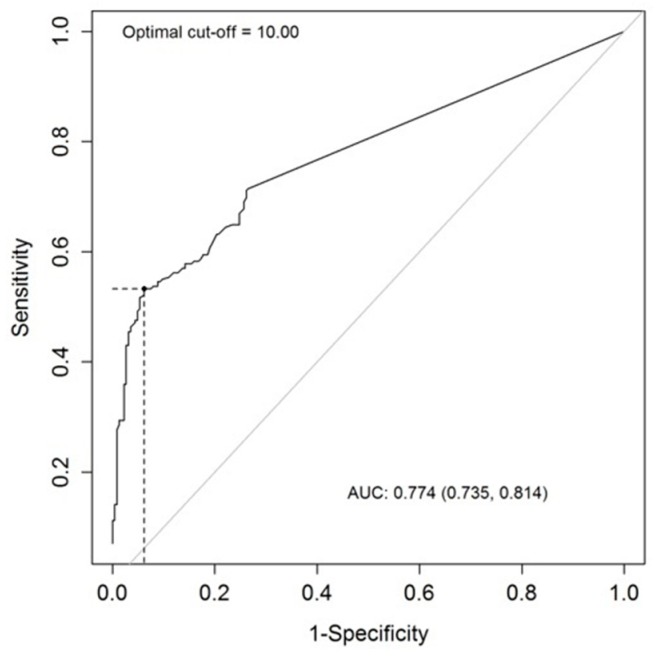
Analysis of the predictive ability—receiver operating (ROC) curve analysis for UBC^®^ Rapid at cut-off value 10.0 µg/L with AUC 0.774 for the whole population.

**Figure 2:**
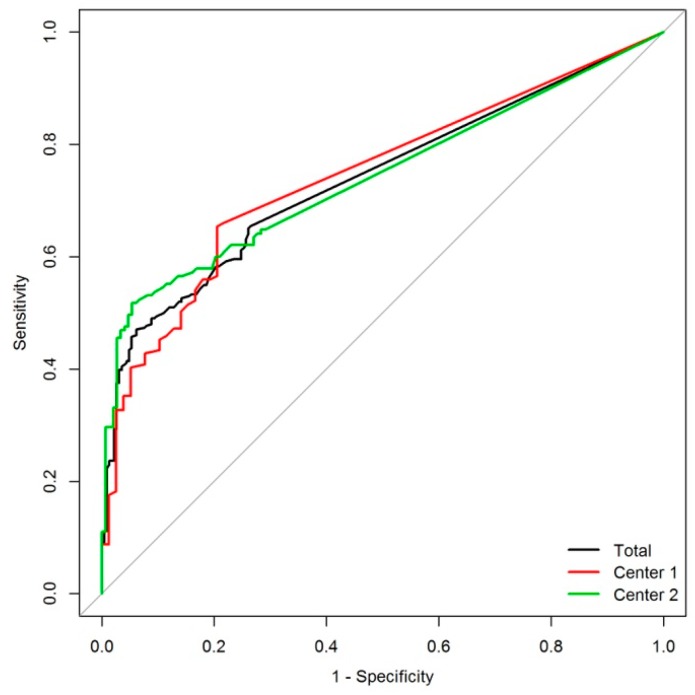
Analysis of the predictive ability—ROC curve analysis for UBC^®^ Rapid at cut-off value 10.0 µg/L for the center 1 (red), center 2 (green), and whole population (black). *p*-value for comparison between centers = 0.874.

**Figure 3:**
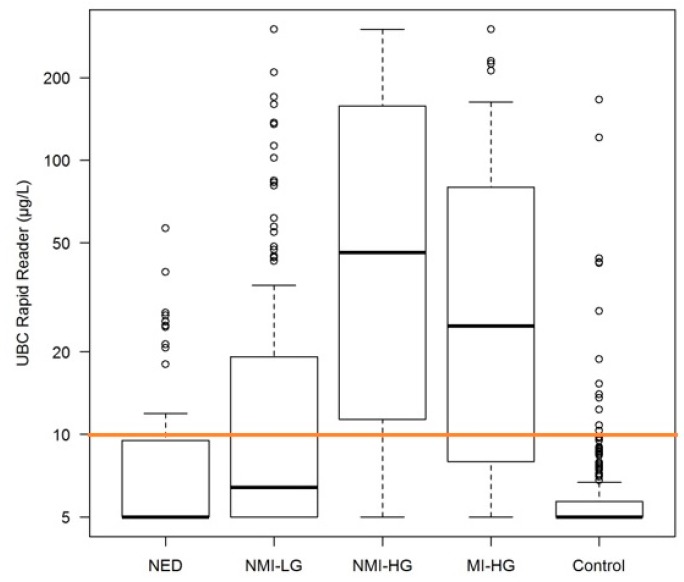
Box plot for non-evidence of disease (NED), non-muscle-invasive low grade (NMI-LG), non-muscle-invasive high grade (NMI-HG), muscle-invasive high grade (MI-HG), control. Orange line for cut-off at 10 µg/L.

We could show that elevated concentrations of UBC^®^ *Rapid* Test are detectable in urine of bladder cancer patients (Table 1 and Table 2). Elevated levels of UBC^®^ *Rapid* Test in urine are higher in patients with bladder cancer in comparison to the control group. In 134 NMI-LG tumors the mean value of UBC^®^ *Rapid* Test was 30.9 µg/L, for NMI-HG tumors 95.5 µg/L, for MI-HG tumors 66.9 µg/L, for NED patients 10.0 µg/L, and for the healthy individuals 7.7 µg/L. Elevated levels of UBC^®^ *Rapid* Test in urine are statistically significantly higher in patients with bladder cancer in comparison to the control group (*p* < 0.0001). The high-risk group showed a markedly higher UBC^®^ *Rapid* signal than the low-risk group. The area under the curve (AUC) of the quantitative UBC^®^ *Rapid* Test using the optimal threshold obtained by receiver operating characteristics curve (ROC) analysis (cut-off 10.0 µg/L) was 0.774 as shown in Figure 1. ROC analyses of patients from center 1, center 2, and all patients together is shown in Figure 2, and demonstrated very similar outcomes. Figure 3 shows the distribution of UBC^®^ *Rapid* values in boxplots in the different patient groups (overall *p* < 0.001). It shows also that most of the elevated values are definitely higher than the cut-point, especially for NMI-HG tumors.

Sensitivity was calculated as 38.8% for NMI-LG, 75.0% for NMI-HG, and 68.3% for MI-HG bladder cancer, and the UBC^®^ *Rapid* specificity was 93.8% for all calculations. 

Data of sensitivity, specificity, positive, and negative predictive values using a cut-off 10.0 µg/L for UBC^®^ *Rapid* Test including the 95% confidence interval are also listed in Table 1 and Table 2.

The data, which were generated in the two centers separately and reported in Figure 2, show impressively that ROC analysis is very similar and the sensitivity and specificity in both centres demonstrate no significant differences (Table 2). In the clinical data base it is obvious that center 1 has a higher rate of patients with diabetes and the rate of cystoscopies is higher in center 2. Though the rate of nitrite positive urine samples in center 2 is higher, the mean value of leucocytes is similar in both centers. Nevertheless, the results for UBC^©^ *Rapid* Test are very similar in both centers demonstrating the robustness and stability of UBC^®^ *Rapid* Test POC assay.

## 3. Discussion

The main purpose of this multi-center study was to evaluate the clinical usefulness of UBC^®^ *Rapid* Test for diagnosis of bladder cancer with a specific focus on patients with NMI-HG tumors of the urinary bladder compared with healthy individuals. The results of the present study show that cytokeratin concentrations determined by UBC^®^ *Rapid* Test measured by POC reader are statistically significant for patients with bladder cancer and healthy controls. Values of UBC^®^ *Rapid* Test in high-grade tumors are significantly higher than in low-grade tumors, NED patients, and healthy individuals. The AUC as a parameter of diagnostic quality was calculated with 0.774. UBC^®^ *Rapid* Test determined quantitatively could be applied to determine the risk for bladder cancer, but also the risk of having a high-grade tumor with increased risk for recurrence. The need for quantitative urinary markers like UBC^®^ *Rapid* Test had also been published before [11]. The results of this study are showing again high values for UBC^®^ *Rapid* Test especially for patients with high-grade bladder cancer [9,12,13,14]. Following from a previously published study with a high number of samples, the results of this study show that this test could be useful for combination in a diagnostic panel for patients of the high-risk group for bladder cancer. In previous reports of UBC^®^ *Rapid*, a sensitivity of 65% and a specificity of 92% was calculated [15,16]. In the study of Mian et al. [16] only the older version UBC^®^ *Rapid* with only visual evaluation was available. In our study, the new version of UBC^®^ *Rapid* Test as cytokeratin assay was used; an improved lateral flow method resulted in a clearer test and control bands to evaluate. In this study UBC^®^ *Rapid* Test was used with the Omega 100 reader to quantify the results.

Data presented in newer UBC^®^ *Rapid* studies reported a cut-off of 12.3 µg/L, a sensitivity, specificity, PPV, and NPV of 60.7%, 70.1%, 46.8%, and 79.3%, respectively, and with an AUC of 0.68 [9]. According to other previous reported UBC^®^ *Rapid* studies in the literature, the sensitivity of the qualitative UBC^®^ *Rapid* Test ranged from 46.2% to 78.4%, and its specificity from 82.4% to 97.4% [9,12,16,17,18,19,20]. These data concur with our own results, whereas the sensitivity was low with 38.8% for detecting non-muscle invasive low-grade tumors. It increased to 75% for non-muscle invasive high-grade tumors and 68.3% for muscle-invasive tumors at a specificity of 93.8%. Therefore, it achieved the highest sensitivity of a single urinary marker test for detecting high-grade bladder cancer. The diagnostic accuracy of the quantitative UBC^®^ *Rapid* Test POC system has been assessed in just five studies, which reported a sensitivity of 46.6% to 64.5% and a specificity ranging from 70.1% to 86.3%, respectively [9,12,17,19,20]. In this multicenter study, we could measure a high number of samples and the results for UBC^©^ *Rapid* Test are very similar in both study centers. There is no significant difference in the ROC analyses, showing that the test is very stable and reproducible.

Currently, many different urinary POC test systems are available on the market, permitting non-invasive and rapid determination of urinary markers. Their diagnostic accuracy, however, is mostly controversially discussed in a small number of studies [21,22]. The sensitivities are usually higher than those reported for urinary cytology alone, but at a lower specificity [5,22]. Nevertheless, additional costs of urinary markers in surveillance protocols are ultimately not justified [23]. 

According to EAU guidelines, the examination of voided urine to detect cancer cells by cytology has a high sensitivity in high-grade tumors and CIS [2]. The major limitations for cytology are that specimens could be hampered by low cellular yield, urinary tract infections, stones, or intravesical instillations. Regardless, experienced readers can exceed specificity of up to 90% [5,24]. However, negative cytology does not exclude a tumor. As method cytology is subjective, on the other side UBC^®^ *Rapid* Test is an objective method that is standardized and reproducible [22].

The use of those urinary markers for routine follow-up is not recommended in clinical practice by current guidelines and remains a debated issue [22,25]. The use of urine markers is only recommended as an adjunct to cystoscopy in current guidelines [26,27,28]. Other tests like Fluorescence in situ hybridization (FISH) and immunocytology have shown improved sensitivity compared with cytology [29,30,31]. These tests are complex and difficult to perform and they require specialized laboratory facilities. 

It is common that new urine tests are compared with the results of cytology. Across a large number of studies, the results varied a lot. Sensitivity for G1-tumors is lower than 30%, for G2-tumors around 60%, and for G3-tumors 90%. Specificity is around 90–95% [32]. In the study of Ritter et al., UBC^®^ *Rapid* Test was also compared with cytology, showing better results for UBC^®^ *Rapid* Test [9]. One limitation of our study is that we had no comparison to cytology, mainly due to the focus on high-grade bladder cancer. But it is also known that urinary cytology is of limited diagnostic value for detecting low-grade bladder tumors compared to high-grade tumors (up to 84% [33]). In the reported study by Pichler et al., the sensitivity of bladder wash cytology was only 21.4%; the sensitivity of high-grade tumors can reach sensitivities of up to 84% [33]. Urinary cytology had been evaluated in many studies previously, and sensitivities and specificities are limited by the experience of the pathologists. This well-known fact has also been reported in recent references. Furthermore, it could also be interesting to include a combination of different tumor markers into BCa diagnostics. How to combine UBC^®^ *Rapid* with other markers has been shown by Gleichenhagen et al. [13]. In this study a combination of UBC^®^ *Rapid* and survivin increased the sensitivity to 66% with a specificity of 95%. For high-grade tumors, the combination showed a sensitivity of 82% and a specificity of 95%. A combination of both assays confirmed the benefit of using marker panels.

In contrast to dichotomized urinary tests, the quantitative character of the UBC^®^ *Rapid* Test enables risk stratification for bladder cancer based on the absolute UBC^®^ *Rapid* Test value. UBC^®^ *Rapid* Test might not only contribute to improved detection of bladder cancer, but also to improved prediction of high-risk tumors. This has also been shown for other quantitative protein-based urinary tests [34]. An approach to objectify risk stratification should include a number of different parameters including quantitative UBC^®^ *Rapid* Test, grade of haematuria, smoking status, age, and gender for developing a nomogram [35].

Of course, there are many other markers on the market, and genetic testing looks especially promising. Regardless, at the moment these markers are a rapid diagnostic tool too complicated to be included into basic and fast diagnostics. Currently, we still must stick to the proteins when we discuss quick testing. However, the “ideal urine-based marker” for detecting bladder cancer recurrence during surveillance would be rapid, non-invasive, and easy to perform and interpret. Furthermore, the assay should possess not only a high specificity to reduce superfluous cystoscopies on oncological follow-up, but also a high sensitivity so that no patient with low-grade and high-grade bladder cancer will be missed [34].

## 4. Materials and Methods

### 4.1. Patients

For this prospective study, 530 urine samples from bladder cancer patients and healthy controls have been collected between January 2014 and October 2015 at the Department of Urology, HELIOS, Hospital Bad Saarow (study center 1), and Lukas Hospital Neuss (study center 2), Germany. The study was approved by the local Institutional Review Board of national Medical Association Brandenburg (AS 147(bB)/2013). All patients with confirmed bladder cancer underwent cystoscopy, bladder ultrasound, and transurethral resection of bladder tumor in case of abnormal findings. Exclusion criteria were any kind of mechanical manipulation (cystoscopy, transrectal ultrasound, and catheterization) within 10 days before urine sampling. Other exclusion criteria were benign prostate enlargement, urolithiasis, other tumor diseases, severe infections, and pregnancy. All these criteria could influence the test to produce false positive results. Less than 10% of the possible study cohort had to be excluded based on exclusion criteria.

### 4.2. Procedure

Voided urine samples were collected in a sterile plastic container and subsequently processed. Urine samples were analyzed by the UBC^®^ *Rapid* Test (Concile GmbH, Freiburg/Breisgau, Germany). All tests were carried out as recommended by the manufacturer’s instructions. The presence of a test band after 10 minutes of incubation was checked. After visual evaluation, the test cartridges were analyzed by the photometric point-of-care (POC) system Concile Omega 100 reader (Concile GmbH, Freiburg/Breisgau, Germany) for quantitative analysis. The cut-off value used for calculation of statistical parameters was based upon the evaluation of the receiver operating characteristics curve (ROC) and defined as 10.0 µg/L. The Omega 100 reader illuminates the test field with a complementary colored light to reduce interference in the analysis. The built-in charge-coupled device–matrix sensor takes a photograph of the light reflected, which is analyzed by the device. 

### 4.3. Statistical Analysis

All statistical analyses have been performed using R version 3.2.3 (R Core Team (2015). R: A language and environment for statistical computing. R Foundation for Statistical Computing, Vienna, Austria. URL https://www.R-project.org/). Data are presented descriptively using means and standard deviations for numerical variables and absolute and relative frequencies for categorical variables. Comparison between groups at baseline has been performed using analysis of variance (ANOVA) for numerical variables and chi-square tests for categorical variables. 

The predictive ability of UBC^®^ *Rapid* Test measurements to detect bladder cancer was evaluated using Receiver Operating Characteristics (ROC) analysis, where the optimal cut-point was determined using the Youden index [36]. Sensitivity, specificity, positive, and negative predictive value was then calculated for the optimal cut-off and presented with exact 95% confidence intervals.

### 4.4. Ethics

The study was performed according to the Declaration of Helsinki. The study was approved by the local Institutional Review Board of National Medical Association Brandenburg (No. AS 147(bB)/2013 dated by 17 November 2013). Written and informed consent was obtained from each participant.

## 5. Conclusions

Elevated values of UBC^®^ *Rapid* Test in urine are higher in patients with non-muscle invasive high-grade bladder cancer in comparison to low-grade tumors and the healthy control group. Sensitivity for non-muscle invasive high-grade tumors is very high with 75% at a specificity of 93.8%. Thus, UBC^®^ *Rapid* Test has the potential to be a more sensitive and specific urinary protein biomarker to identify patients with high-grade tumors that are difficult to detect in cystoscopy. Results for UBC^®^ *Rapid* Test in both study centers of the present multicenter study are very similar and reproducible. UBC^®^ *Rapid* Test is standardized and calibrated, and thus independent of use, batch of test, as well as study site. UBC^®^ *Rapid* Test should be added in the diagnostics and follow-up for NMI-HG tumors of urinary bladder cancer, though cystoscopy is still an important part of monitoring of bladder cancer.

## Figures and Tables

**Table 1:** Patient characteristics and results of UBC^®^ *Rapid.* Abbreviations: non-muscle-invasive low grade (NMI-LG), non-muscle-invasive high grade (NMI-HG), muscle-invasive high grade (MI-HG), non-evidence of disease (NED), positive predictive value (PPV), negative predictive value (NPV).

	NMI-LG	NMI-HG	MI-HG	NED	Control	*p*-Value
***n***	134	48	60	62	226	
**Age** Mean (SD) Median Range	71.0 (11.9) 73.5 26–92	73.8 (10.6) 75 51–94	73.6 (10.0) 74.5 52–98	70.8 (11.4) 72 46–88	68.9 (12.2) 71 31–93	0.036
**Sex** Female (%) Male (%)	34 (25.4) 100 (74.6)	6 (12.5) 42 (87.5)	19 (31.7) 41 (68.3)	10 (16.1) 52 (83.9)	70 (31.0) 156 (69.0)	0.021
**Diabetes**	25 (18.7%)	8 (16.7%)	12 (20%)	10(16.1%)	34 (15%)	0.849
**Erythrocyte (urine dipstick)**	83 (61.9%)	44 (91.7%)	58 (96.7%)	38 (61.3%)	73 (32.3%)	<0.001
**Leucocytes (urine dipstick)** Mean (SD) Median Range	84.6 (172.6) 0 0–500	109.9 (167.6) 25 0–500	229.7 (228.1) 100 0–500	127.8 (206.6) 20 0–500	64.3 (150.3) 0 0–500	<0.001
**Nitrite pos.**	5 (3.7%)	5 (10.4%)	8 (13.3%)	3 (4.8%)	12 (5.3%)	0.085
**Cystoscopy**	100 (74.6%)	33 (68.8%)	42 (70%)	49 (79%)	36 (15.9%)	<0.001
**UBC (µg/L)** Mean (SD) Median Range	30.9 (63.4) 6.4 5–300	95.5 (104.6) 46 5–300	66.9 (90.1) 24.85 5–300	10 (9.79) 5 5–56.5	7.7 (13.92) 5 5–166	<0.001
Sensitivity Specificity PPV NPV	38.8% 93.8% 78.8% 72.1%	75.0% 93.8% 72.0% 94.6%	68.3% 93.8% 74.6% 94.6%	22.6% 93.8% 50% 81.5%		

**Table 2:** Patient characteristics and results of UBC^®^ *Rapid* separated for center 1 and center 2.

	NMI-LG		NMI-HG		MI-HG		NED		Control		*p*-Value	
	Center 1	Center 2	Center 1	Center 2	Center 1	Center 2	Center 1	Center 2	Center 1	Center 2	Center 1	Center 2
***n***	78	56	26	22	25	35	30	32	78	148		
**Age** Mean (SD) Median Range	71.1 (11.6) 74 33–90	70.8 (12.4) 72 26–92	74.9 (11.6) 78.5 52–94	72.5 (9.4) 75 51–92	74.2 (11.0) 75 52–98	73.2 (9.3) 74 53–88	73.0 (8.8) 73.5 46–88	68.8 (13.1) 70.5 46–88	67.6 (12.64) 69.5 31–86	69.6 (11.94) 71.5 33–93	0.042	0.554
**Gender ** Female (%) Male (%)	20 (25.6) 58 (74.4)	14 (25.0) 42 (75.0)	4 (15.4) 22 (84.6)	2 (9.1) 20 (90.9)	6 (24.0) 19 (76.0)	13 (37.1) 22 (62.9)	3 (10.0) 27 (90.0)	7 (21.9) 25 (78)	23 (29.5) 55 (70.5)	47 (31.8) 101 (68.2)	0.220	0.127
**Diabetes**	22 (28.2%)	3 (5.4%)	6 (23.1)	2 (9.1%)	6 (24%)	6 (17.1%)	5 (16.7%)	5 (15.6%)	13 (16.7%)	21 (14.2%)	0.469	0.337
**Erythrocyte pos.**	45 (57.7%)	38 (67.9%)	24 (92.3%)	20 (91.0%)	24 (96.0%)	34 (97.1%)	17 (56.7%)	21 (65.6%)	34 (43.6%)	39 (26.4%)	<0.001	<0.001
**Leucocytes** Mean (SD) Median Range	79.17 (165.2) 0 0–500	92.39 (183.8) 0 0–500	142.3 (182.04) 100 0–500	71.6 (143.4) 25 0–500	249.0 (229.41) 100 0–500	215.4 (229.5) 100 0–500	142.50 (220.1) 25 0–500	113.55 (195.2) 0 0–500	76.6 (165.60) 0 0–500	57.7 (141.68) 0 0–500	<0.001	<0.001
**Nitrite pos.**	3 (3.8%)	2 (3.6%)	4 (5.1%)	1 (1.8%)	2 (8%)	6 (17.1%)	1 (3.3%)	2 (6.3%)	3 (3.8%)	9 (6.1%)	0.199	0.185
**Cystoscopy**	47 (60.3%)	53 (94.6%)	11 (42.3%)	22 (100%)	11 (44%)	31 (88.6%)	20 (66.7%)	29 (90.6%)	16 (20.5%)	20 (13.5%)	<0.001	<0.001
**UBC [µg/L]** Mean (SD) Median Range	21.1 (43.7) 6.5 5–300	44.6 (81.9) 6.2 5–300	83.9 (94.8) 41.4 5–300	109.3 (115.8) 59.4 5–300	61.4 (80.9) 28.9 5–300	70.9 (97.1) 20.7 5–300	7.48 (7.06) 5 5–39.1	12.37 (11.4) 6.5 5–56.5	7.8 (13.9) 5 5–121	7.6 (14.0) 5 5–166	<0.001	<0.001
Sensitivity Specificity PPV NPV	38.5% 92.3% 83.3% 60.0%	39.3% 94.6% 73.3% 80.5%	73.1% 92.3% 76.0% 91.1%	77.3% 94.6% 68.0% 96.6%	68.0% 92.3% 73.9% 90.0%	68.6% 94.6% 75.0% 92.7%	6.7% 92.3% 25% 72%	37.5% 94.6% 60% 87.5%				
